# Relationship Between the 25-Question Geriatric Locomotive Function Scale and Falls: A One-Year Longitudinal Observational Study of 1,173 Healthy Community-Dwelling Residents Aged 65 and Older

**DOI:** 10.7759/cureus.72907

**Published:** 2024-11-03

**Authors:** Takaomi Kobayashi, Tadatsugu Morimoto, Chisato Shumanoe, Rei Ono, Koji Otani, Masaaki Mawatari

**Affiliations:** 1 Department of Orthopaedic Surgery, Saga University, Saga, JPN; 2 Department of Orthopaedics, Saga University, Saga, JPN; 3 Department of Pharmacy, Saga University, Saga, JPN; 4 Department of Physical Activity Research, National Institute of Health and Nutrition, National Institutes of Biomedical Innovation, Health and Nutrition, Osaka, JPN; 5 Department of Orthopaedic Surgery, Fukushima Medical University, Fukushima, JPN

**Keywords:** 25-question geriatric locomotive function scale, fall, health check, japan, locomotive syndrome

## Abstract

Introduction

This study aimed to explore the relationship between the 25-question Geriatric Locomotive Function Scale (GLFS-25) score (i.e., total score and domain scores) and falls (i.e., history with or without falls and frequency of falls).

Methodology

We conducted a one-year longitudinal observational study involving 1,173 healthy community-dwelling residents aged ≥65 years who attended a basic health checkup in Minami-Aizu Town and Tadami Town, Fukushima, Japan, from 2016 to 2017. The following clinical information was collected: age, sex, body mass index, smoking status, alcohol consumption, living situation, metabolic syndrome, physical activity, and GLFS-25 score during the participants’ health check in 2016. The GLFS-25 measures various domains, including body pain, movement-related difficulties, usual care, social activities, and anxiety. Participants were diagnosed with locomotive syndrome (LS) based on their GLFS-25 total scores: Non-LS (0-6 points), LS-1 (7-15 points), LS-2 (16-23 points), and LS-3 (24-100 points). We assessed the annual occurrence of falls during the participants’ health check in 2017 and the monthly frequency of falls. Student's t-test, Mann-Whitney's U test, and Fisher's exact test were performed to compare parameters between fallers and non-fallers. To examine the association between the annual occurrence of falls and the diagnosis of LS, a multivariate logistic regression analysis was performed to calculate adjusted odds ratios (ORs), controlled based on the clinical information. To assess the association between the monthly frequency of falls and GLFS-25 scores, a multivariate regression analysis was performed to calculate the adjusted standardized partial regression coefficient (β), controlled based on the clinical information.

Results

Fallers were significantly older (p < 0.001), had a higher body mass index (p = 0.034), and had higher GLFS-25 total scores (p < 0.001) than non-fallers. In the multiple logistic regression analysis, falls were significantly associated with LS-1 or more (OR = 2.32, p < 0.001), LS-2 or more (OR = 2.72, p < 0.001), and LS-3 or more (OR = 2.99, p < 0.001). Furthermore, the annual occurrence of falls was significantly associated with GLFS-25 body pain (OR = 1.94, p = 0.012) and anxiety scores (OR = 2.09, p = 0.021). In the multiple regression analysis, the monthly frequency of falls was significantly associated with the GLFS-25 total score (β = 0.29, p < 0.001). The monthly frequency of falls was also significantly associated with GLFS-25 domain scores, including body pain score (β = 0.23, p < 0.001), movement-related difficulty score (β = 0.21, p < 0.001), usual care score (β = 0.18, p < 0.001), social activity score (β = 0.26, p < 0.001), and anxiety score (β = 0.22, p < 0.001).

Conclusion

Our findings emphasize the importance of fall prevention in individuals with LS-1 and suggest that the GLFS-25 total score may predict recurrent falls. Our study first provides valuable evidence regarding the relationship between the GLFS-25 (total score and domain scores) and falls. The monthly frequency of falls was correlated with the total GLFS-25 score and all GLFS-25 domain scores. However, the annual occurrence of falls was found to have no correlation with anything other than the GLFS-25 domain scores regarding physical pain and anxiety. Therefore, further investigations are needed.

## Introduction

Japan has had the world’s largest aged population since 2005 [1], and the percentage of people aged ≥65 years surged by 28.6% in 2020 [2]. Projections indicate that this trend will persist, with the elderly population expected to reach 34.8% by 2040 [3]. Consequently, the occurrence of falls among older adults and the resulting fragility fractures have become serious societal concerns. For example, it is estimated that the number of osteoporotic hip fractures due to falls will reach 226,000 in 2020 and is expected to rise to 304,000 by 2040 [4]. Additionally, the projected number of osteoporotic vertebral fractures due to falls is set to increase from 489,000 in 2020 to 555,000 in 2040 [4]. These factors contribute to high mortality, morbidity, immobility, and early nursing home admissions [5]. Therefore, early detection and prevention of falls are important.

Locomotive syndrome (LS) is characterized by reduced mobility due to age-related muscle weakness or musculoskeletal diseases [6]. LS risk tests were designed to evaluate the extent of LS severity, including the 25-question Geriatric Locomotive Function Scale (GLFS-25) [6,7], a two-step test [6], and a stand-up test [6]. Recent studies have explored the association between the risk of LS and falls [8-15]. Among the LS risk tests, the GLFS-25 is commonly utilized in clinical and research contexts [9,12,13], and it is the only LS risk test that may be associated with falls. However, variations in subject definitions and fall criteria have led to an ongoing debate regarding their relationship.

To date, no studies have conducted detailed evaluations, such as the GLFS-25 domain score (i.e., body pain, movement-related difficulties, usual care, social activities, and anxiety [7]), in conjunction with the frequency of falls; recurrent falls may increase the risk of serious injury [9]. Clarifying these factors could lead to the elucidation of factors related to the occurrence of falls and contribute to simple fall prediction (using certain questions) and the prevention of fall-related osteoporotic fractures. Hence, this study aimed to explore the relationships between the GLFS-25 scores (i.e., total score and domain scores) and falls (i.e., the history and frequency of falls).

## Materials and methods

Study design

The present study adhered to the Strengthening the Reporting of Observational Studies in Epidemiology (STROBE) guidelines [16] and received Institutional Ethics Committee approval. We conducted a one-year longitudinal observational study involving healthy community-dwelling residents aged ≥65 years who attended a basic health checkup in Minami-Aizu Town and Tadami Town, Fukushima Prefecture, Japan, from 2016 to 2017. The participants provided their written informed consent for interviews and participation in the study. Detailed information from this study in 2016 has been described previously [17-19].

**Figure 1:**
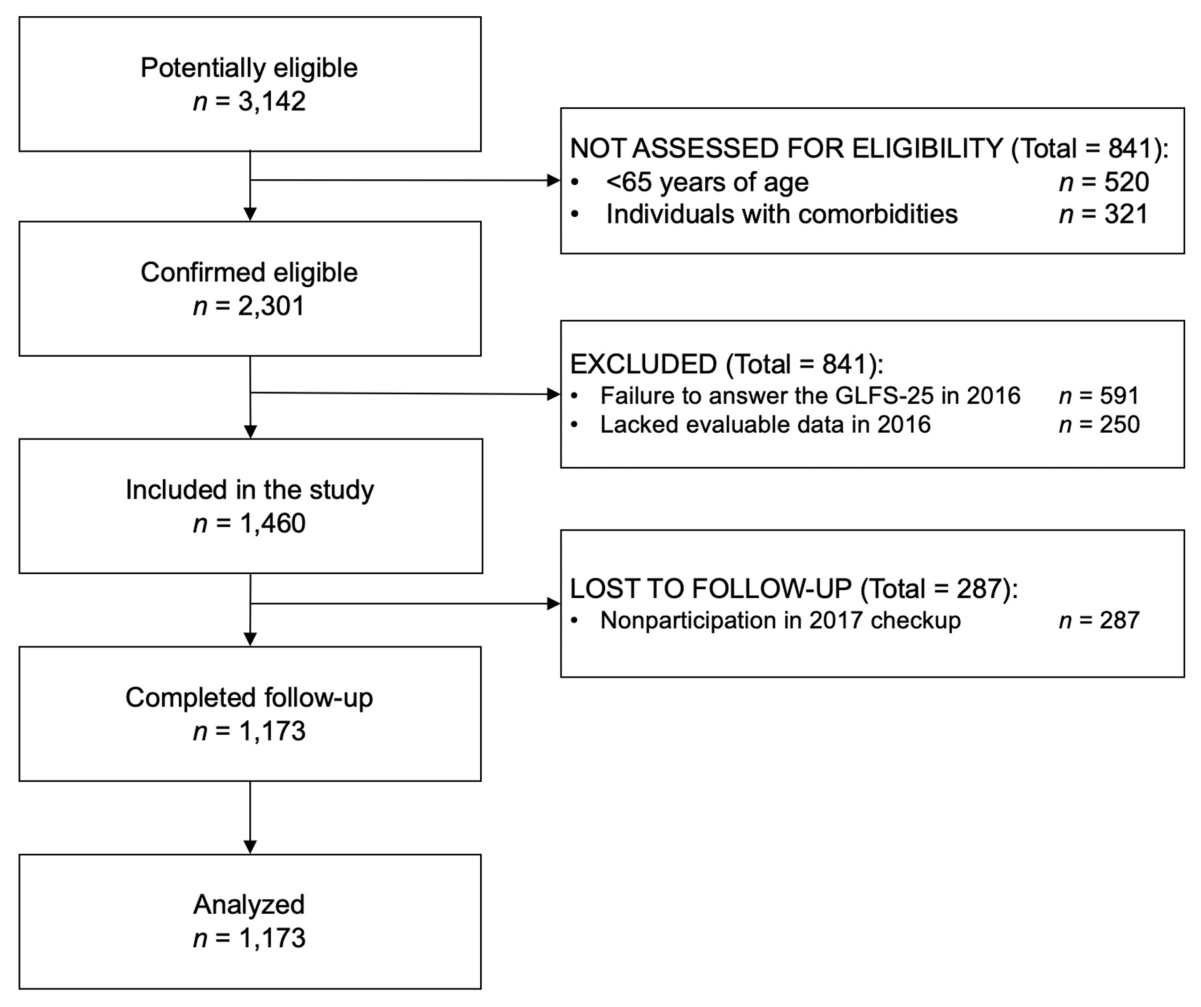
Study flow diagram

From the initial pool of 3,142 potentially eligible participants, 1,173 individuals (511 males, 662 females) with a median age of 72.0 years were included in this study (Figure 1). Patients aged <65 years (n = 520) and those with comorbidities (n = 321) were excluded. Additionally, participants who did not complete all items of the GLFS-25 (n = 591), those without evaluable data (n = 250), and those who were lost to follow-up (n = 287) were excluded. The follow-up rate was 80.3% (1,173/1,460 individuals).

Fall assessment

Falls were assessed during the subsequent year’s health checkups in 2017. Participants were asked, “Have you fallen down in the past year?” Those who answered “yes” were classified as fallers, while those who answered “no” were classified as non-fallers [9,12]. We conducted additional investigations into the frequency of falls during the month preceding the basic health checkup.

LS assessment

The assessment of LS was conducted during the initial health check in 2016 using the GLFS-25 (Table 1) [7], which consists of 25 items graded on a five-point scale (0-4 points). The scale measures various domains, including body pain (items 1-4), movement-related difficulties (items 5-7), usual care (items 8-11 and 14), social activities (items 12, 13, and 15-23), and anxiety (items 24 and 25) [7]. Participants were diagnosed with LS based on their GLFS-25 total scores: Non-LS (0-6 points), LS-1 (7-15 points), LS-2 (16-23 points), and LS-3 (24-100 points) [6,7].

**Table 1:** GLFS-25 questionnaire items GLFS-25: 25-question Geriatric Locomotive Function Scale

Questionnaire items	Domain
1. Have you had any pain (including numbness) in your neck or upper limbs?	Body pain
2. Have you had any pain in your back, lower back, or buttocks?	Body pain
3. Have you had any pain (including numbness) in your lower limbs?	Body pain
4. To what extent has it been painful to move your body in daily life?	Body pain
5. To what extent has it been difficult to get up from a bed or lie down?	Movement-related difficulty
6. To what extent has it been difficult to stand up from a chair?	Movement-related difficulty
7. To what extent has it been difficult to walk inside the house?	Movement-related difficulty
8. To what extent has it been difficult to put on and take off a shirt?	Usual care
9. To what extent has it been difficult to put on and take off trousers and pants?	Usual care
10. To what extent has it been difficult to use the toilet?	Usual care
11. To what extent has it been difficult to wash your body in the bath?	Usual care
12. To what extent has it been difficult to go up and down the stairs?	Social activities
13. To what extent has it been difficult to walk briskly?	Social activities
14. To what extent has it been difficult to keep yourself neat?	Usual care
15. How far can you continuously walk without resting?	Social activities
16. To what extent has it been difficult to go out to visit neighbors?	Social activities
17. To what extent has it been difficult to carry objects weighing 2 kg?	Social activities
18. To what extent has it been difficult to go out using public transportation?	Social activities
19. To what extent have simple tasks and housework been difficult?	Social activities
20. To what extent have load-bearing tasks and housework been difficult?	Social activities
21. To what extent has it been difficult to perform sports activities?	Social activities
22. Have you felt restricted from meeting your friends?	Social activities
23. Have you felt restricted from joining social activities?	Social activities
24. Have you ever felt anxious about falls in your house?	Anxiety
25. Have you ever felt anxious about being unable to walk in the future?	Anxiety

Participants’ characteristics assessment

Participants’ characteristics were evaluated during the initial health checkup in 2016. This assessment included age, sex, body mass index, smoking status (current smoker, former smoker, or non-smoker), alcohol consumption (everyday, sometimes, or no/rare), living situation, metabolic syndrome (MS) assessment (MS, pre-MS, or non-MS), and physical activity (low, middle, or high).

MS was defined as having two or more components in addition to visceral obesity [20,21]. Pre-MS was defined as the presence of a component in addition to visceral obesity. Non-MS was defined as not meeting the criteria for diagnosis of MS or pre-MS. Visceral obesity was defined as a waist circumference ≥85 cm for men and ≥90 cm for women. Components were defined as follows: high blood pressure (systolic blood pressure ≥130 mmHg and/or diastolic blood pressure ≥85 mmHg), dyslipidemia (high-density lipoprotein (HDL) cholesterol <40 mg/dL and/or triglyceride ≥150 mg/dL), and hyperglycemia (HbA1c ≥6.0%). Individuals receiving treatment for hypertension, dyslipidemia, or diabetes mellitus were also included in the above definitions [20,21]. The physical activity level was estimated using the International Physical Activity Questionnaire short form (IPAQ-SF), a validated questionnaire consisting of nine questions regarding the time spent engaged in vigorous- and moderate-intensity activities, walking, and sedentary activity in a usual week [22]. In this study, the sum of vigorous (8 metabolic equivalents (METs)), moderate (4 METs), and walking (3.3 METs) activities was calculated as MET hours/week. According to the IPAQ (2005) guidelines [22], the IPAQ-SF score was classified as categorical data: <10 MET-hours/week (low), ≥10, <50 MET-hours/week (middle), and ≥50 MET-hours/week (high).

Statistical analyses

We assessed the normality of the distribution of quantitative data using the Shapiro-Wilk test. Subsequently, we employed Student's t-test to compare quantitative data that showed a normal distribution, and Mann-Whitney's U test to compare quantitative data that did not show a normal distribution between fallers and non-fallers. Qualitative data were compared between the two groups using Fisher's exact test.

To examine the association between the annual occurrence of falls and the diagnosis of LS, we conducted a logistic regression analysis to calculate the odds ratios (ORs). In this analysis, the annual occurrence of falls (0: absent, 1: present) served as the dependent variable, while the diagnosis of LS (0: absent, 1: present) was the independent variable. In addition, the GLFS-25 domain scores (body pain (0: 0-7 points, 1: 8-16 points), movement-related difficulties (0: 0-5 points, 1: 6-12 points), usual care (0: 0-9 points, 1: 10-20 points), social activities (0: 0-21 points, 1: 22-44 points), and anxiety (0: 0-3 points, 1: 4-8 points)) served as the independent variables. GLFS-25 domain scores were categorized based on the midpoint of their possible values. To assess the association between the monthly frequency of falls and GLFS-25 scores, a regression analysis was used to calculate the standardized partial regression coefficient (β). In this analysis, the monthly frequency of falls (times, continuous) served as the dependent variable, while the GLFS-25 scores (points, continuous) were the independent variable. In addition, the GLFS-25 domain scores (body pain (points, continuous), movement-related difficulties (points, continuous), usual care (points, continuous), social activities (points, continuous), and anxiety (points, continuous)) served as the independent variables. In model 1, no factors were controlled for in this study. In model 2, factors with p < 0.20 in the comparison analyses, in addition to age, sex, and body mass index, were controlled based on previous studies [8-15]. In model 3, smoking status, alcohol consumption, living alone, MS assessment, and physical activity were controlled, in addition to age, sex, and body mass index, since apparent associations may exist despite nonsignificant differences in the comparison analysis and previous studies [8-15].

The significance level was set at 0.05, and analyses were conducted using JMP® Pro 16 (SAS Institute, Cary, North Carolina, USA).

## Results

Relationship between the annual occurrence of falls and the GLFS-25

The comparison analyses showed that fallers were significantly older (median: 73.0 years vs. 72.0 years, p < 0.001), had a slightly higher body mass index (mean: 24.0 kg/m² vs. 23.6 kg/m², p = 0.034), and had higher GLFS-25 total scores (median: 10 points vs. 5 points, p < 0.001) compared to non-fallers (Table 2). In model 3, the annual occurrence of falls was significantly associated with LS-1 or more (adjusted OR = 2.32, p < 0.001), LS-2 or more (adjusted OR = 2.72, p < 0.001), and LS-3 or more (adjusted OR = 2.99, p < 0.001) (Table 3). Furthermore, in model 3, the annual occurrence of falls was significantly associated with the GLFS-25 body pain (adjusted OR = 1.94, p = 0.012) and anxiety scores (adjusted OR = 2.09, p = 0.021) (Table 4).

**Table 2:** Characteristics of the study participants and comparisons between fallers and non-fallers ^a^Values are shown as the mean ± standard deviation and were compared using Student’s t-test; ^b^Values are shown as the median (minimum-maximum) and were compared using the Mann-Whitney U test; ^c^Values are shown as the number (percentage) and were compared using Fisher's exact test *Statistically significant value (p < 0.05) between groups GLFS-25: 25-question Geriatric Locomotive Function Scale; LS: Locomotive syndrome; MS: Metabolic syndrome

	Overall (n = 1,173)	Faller (n = 252)	Non-faller (n = 921)	p-value
Age, years	72.0 (65.0-93.0)	73.0 (65.0-93.0)	72.0 (65.0-90.0)	0.005^b^*
Age category, n (%)				0.054^c^
65-69 years	425 (36.2)	75 (29.8)	350 (38.0)	-
70-74 years	329 (28.0)	77 (30.6)	252 (27.4)	
≥75 years	419 (35.7)	100 (39.7)	319 (34.6)	
Female, n (%)	662 (56.4)	152 (60.3)	510 (55.4)	0.161^c^
Body mass index, kg/m^2^	23.7 ± 3.1	24.0 ± 3.0	23.6 ± 3.1	0.034^a^*
Smoking status, n (%)				0.304^c^
Current smoker	87 (7.4)	13 (5.2)	74 (8.0)	-
Former smoker	309 (26.3)	68 (27.0)	241 (26.2)	
Non-smoker	777 (66.2)	171 (67.8)	606 (65.8)	
Alcohol consumption, n (%)				0.921^c^
Everyday	309 (26.3)	64 (25.4)	245 (26.6)	-
Sometimes	213 (18.2)	47 (18.7)	166 (18.0)	
No/rare	651 (55.5)	141 (55.9)	510 (55.4)	
Living alone, n (%)	185 (15.8)	45 (17.9)	140 (15.2)	0.305^c^
MS assessment, n (%)				0.260^c^
Non-MS	750 (63.9)	151 (59.9)	599 (65.0)	-
Pre-MS	158 (13.5)	35 (13.9)	123 (13.4)	
MS	265 (22.6)	66 (26.2)	199 (21.6)	
Physical activity, n (%)				0.358^c^
Low	392 (33.4)	93 (36.9)	299 (32.5)	-
Middle	379 (32.3)	74 (29.4)	305 (33.1)	
High	402 (34.2)	85 (33.7)	317 (34.4)	
GLFS-25 total score, points	5 (0-63)	10 (0-63)	5 (0-59)	<0.001^b^*
GLFS-25 domain score, points				
Body pain	2 (1-4)	4 (2-6)	2 (1-4)	<0.001^b^*
Movement-related difficulties	0 (0-0)	0 (0-2)	0 (0-0)	<0.001^b^*
Usual care	0 (0-0)	0 (0-1)	0 (0-0)	<0.001^b^*
Social activities	2 (0-6)	4 (1-10)	2 (0-5)	<0.001^b^*
Anxiety	0 (0-1)	1 (0-2)	0 (0-1)	<0.001^b^*
Diagnosis of LS, n (%)				<0.001^c^*
Non-LS	641 (54.6)	92 (36.5)	549 (59.6)	-
LS-1	330 (28.1)	80 (31.7)	250 (27.1)	
LS-2	87 (7.4)	29 (11.5)	58 (6.3)	
LS-3	115 (9.8)	51 (20.2)	64 (6.9)	

**Table 3:** Logistic regression analyses using the annual occurrence of falls as dependent variables, and diagnosis of LS as independent variables ^a^Model 1: unadjusted; ^b^Model 2: adjusted for age, sex, and body mass index; ^c^Model 3: adjusted for age, sex, body mass index, smoking status, alcohol consumption, living alone, MS, and physical activity. *Statistically significant value (p < 0.05) between groups LS: Locomotive syndrome; OR: Odds ratio; MS: Metabolic syndrome

		Model 1^a^		Model 2^b^		Model 3^c^	
		OR	p-value	OR	p-value	OR	p-value
LS-1 or more	Absent	1.00 (Reference)	-	1.00 (Reference)	-	1.00 (Reference)	-
	Present	2.57	<0.001	2.34	<0.001	2.32	<0.001*
LS-2 or more	Absent	1.00 (Reference)	-	1.00 (Reference)	-	1.00 (Reference)	-
	Present	3.05	<0.001	2.7	<0.001	2.72	<0.001*
LS-3 or more	Absent	1.00 (Reference)	-	1.00 (Reference)	-	1.00 (Reference)	-
	Present	3.4	<0.001	2.89	<0.001	2.99	<0.001*

**Table 4:** Logistic regression analyses using the annual occurrence of falls as a dependent variable, and GLFS-25 domain scores as independent variables ^a^Model 1: unadjusted; ^b^Model 2: adjusted for age, sex, and body mass index; ^c^Model 3: adjusted for age, sex, body mass index, smoking status, alcohol consumption, living alone, MS, and physical activity *Statistically significant value (p < 0.05) between groups GLFS-25: 25-question Geriatric Locomotive Function Scale; OR: Odds ratio; MS: Metabolic syndrome

		Model 1^a^		Model 2^b^		Model 3^c^	
		OR	p-value	OR	p-value	OR	p-value
Body pain	0-7 points	1.00 (Reference)	-	1.00 (Reference)	-	1.00 (Reference)	-
	8-16 points	2.24	0.003	1.97	0.01	1.94	0.012*
Movement-related difficulties	0-5 points	1.00 (Reference)	-	1.00 (Reference)	-	1.00 (Reference)	-
	6-12 points	2.6	0.055	2.16	0.126	2.19	0.127
Usual care	0-9 points	1.00 (Reference)	-	1.00 (Reference)	-	1.00 (Reference)	-
	10-20 points	1.22	0.808	1.02	0.979	1.04	0.965
Social activities	0-21 points	1.00 (Reference)	-	1.00 (Reference)	-	1.00 (Reference)	-
	22-44 points	1.62	0.296	1.24	0.641	1.21	0.686
Anxiety	0-3 points	1.00 (Reference)	-	1.00 (Reference)	-	1.00 (Reference)	-
	4-8 points	2.75	<0.001	2.11	0.017	2.09	0.021*

Relationship between the monthly frequency of falls and the GLFS-25 scores

The average monthly fall rate was 0.3. Eight participants experienced four falls, 24 participants experienced three falls, 82 participants experienced two falls, 138 participants experienced one fall, and 921 participants experienced no falls. In model 3, the monthly frequency of falls was significantly associated with the GLFS-25 total score (adjusted β = 0.29, p < 0.001) and domain scores, including body pain score (adjusted β = 0.23, p < 0.001), movement-related difficulty score (adjusted β = 0.21, p < 0.001), usual care score (adjusted β = 0.18, p < 0.001), social activity score (adjusted β = 0.26, p < 0.001), and anxiety score (adjusted β = 0.22, p < 0.001) (Table 5).

**Table 5:** The regression analyses using the monthly frequency of falls as a dependent variable, and GLFS-25 scores as independent variables ^a^Model 1: unadjusted; ^b^Model 2: adjusted for age, sex, and body mass index; ^c^Model 3: adjusted for age, sex, body mass index, smoking status, alcohol consumption, living alone, MS, and physical activity *Statistically significant value (p < 0.05) between groups GLFS-25: 25-question Geriatric Locomotive Function Scale; β: Standardized partial regression coefficient; MS: Metabolic syndrome

	Model 1^a^		Model 2^b^		Model 3^c^	
	β	p-value	β	p-value	β	p-value
Total score	0.29	<0.001	0.29	<0.001	0.29	<0.001*
Domain scores						
Body pain	0.25	<0.001	0.23	<0.001	0.23	<0.001*
Movement-related difficulties	0.22	<0.001	0.21	<0.001	0.21	<0.001*
Usual care	0.2	<0.001	0.18	<0.001	0.18	<0.001*
Social activities	0.26	<0.001	0.25	<0.001	0.26	<0.001*
Anxiety	0.26	<0.001	0.22	<0.001	0.22	<0.001*

## Discussion

Our main findings can be summarized as follows: (1) fallers had significantly higher GLFS-25 total scores than non-fallers; (2) the annual occurrence of falls was significantly related to diagnoses of LS-1 or more, categorized by the GLFS-25; (3) the annual occurrence of falls was significantly associated with the GLFS-25 body pain and anxiety scores; and (4) there was a significant association between the monthly frequency of falls and the GLFS-25 scores among community-dwelling residents aged ≥65 years.

We observed that the annual occurrence of falls was associated with significantly higher GLFS-25 total scores. This positive correlation between GLFS-25 total scores and the likelihood of falling is consistent with the GLFS total score and corresponding fall rate in previous studies [10,12,13]. In studies conducted by Arai et al. [12], Iida et al. [13], and Kimura et al. [10], the GLFS-25 total scores were 5.7 ± 6.7 with a 13.5% fall prevalence, 8.2 ± 9.2 with a 15.9% fall prevalence, and 30.2 ± 22.7 with a 28.3% fall prevalence. Thus, a higher GLFS-25 total score is associated with an increased probability of falling.

We discovered a significant association between future falls and diagnoses of LS-1 or more. Our findings align with those of Arai et al. [12], who observed that fallers had significantly higher GLFS-25 total scores than did non-fallers (mean: 8.0 vs. 5.4; p = 0.04). In contrast, other studies reported higher GLFS-25 total scores (ranging from 12.9 to 32.8) among fallers [9,10,13]. This variation may be attributed to differences in the study populations and fall assessment methods. Kimura et al. [9,10] focused on postoperative patients with degenerative cervical myelopathy, while Iida et al. [13] defined falls using the five-item version of the fall risk index. As a result, we determined that falls were significantly associated with diagnoses of LS-1 or more in community-dwelling residents (age: ≥65 years). This underscores the importance of fall prevention strategies, particularly for individuals with a diagnosis of LS-1 or more.

Interestingly, the annual occurrence of falls was significantly associated with GLFS-25 body pain and anxiety scores. Consistent with our findings, previous studies have reported a significant association between falls and chronic pain (including lower and upper limb pain) [10,13,23,24] as well as anxiety [10,13,25]. Therefore, by concentrating on the GLFS-25 body pain and anxiety scores, it will be possible to more effectively assess susceptibility to falls. In other words, alleviating back, knee, or hip pain and addressing anxiety through measures such as the regular administration of oral painkillers, creating an environment to prevent falls at home, using a cane or walker, and providing periodic exercise guidance could contribute to fall prevention. We believe that body pain and anxiety about falling at home, along with concerns about future walking ability, may be useful predictors of annual fall occurrences.

We investigated healthy community-dwelling residents with a median GLFS-25 score of 5.0 and found no significant association between the annual occurrence of falls and GLFS-25 movement-related difficulties, usual care, or social activities scores (Table 4), as well as IPAQ-SF (Table 2). Similarly, other studies explored healthy community-dwelling residents with GLFS-25 scores ranging from 5.8 to 8.2 and also found no significant relationship between falls and objective physical evaluations (e.g., stand-up test [13], two-step test [12,13], grip strength [13], 10 m gait speed [13], timed up and go test [13], five-time sit-and-stand test [12], or one-leg standing time [12]). Kimura et al. [10] investigated postoperative patients with degenerative cervical myelopathy, whose GLFS-25 mean score was 30.2, and found a relationship between falls and GLFS-25 social activity scores (i.e., items 12, 13, 15, 17, and 20) [10]. This discrepancy can be attributed to differences in the study populations; falls and activities of daily living (i.e., movement-related difficulties, usual care, and social activities) may be associated with each other in populations with high GLFS-25 total scores.

We first found a significant association between the monthly frequency of falls and GLFS-25 scores. This finding aligns with previous studies that suggest that individuals who experience frequent falls tend to have higher GLFS-25 scores, although statistical significance was not tested in these studies [9,10]. Therefore, it appears that the GLFS-25 total score not only predicts the presence or absence of falls but may also offer some predictive value regarding the likelihood of recurrent falls. However, in healthy community-dwelling residents of ≥65 years of age, the annual occurrence of falls was found to have no correlation with anything other than the GLFS-25 domain score regarding physical pain and anxiety. Therefore, further investigations are needed.

Limitations of the present study

The present study was associated with several limitations that may affect the generalizability of our findings. First, there may have been a selection bias, as many patients were excluded from the present study. The main cause was the low response rate of the GLFS-25, as reported previously [17,26]. Nevertheless, the follow-up rate of this longitudinal observational study was considered acceptable (≥80%) [27]. Second, it is possible that the use of self-administered questionnaires to evaluate fall history was associated with recall bias. This is why we did not investigate the frequency of falls during the month before the basic health checkup. Further investigation using monthly medical interviews [8], fall diaries [10], and Internet of Things-based health monitoring systems [28] is required to combat this bias. Third, we could not account for cognitive function, which might have influenced the results of the questionnaire survey. Finally, some fall-related factors, including medications and daily environmental risks, were not investigated in this study. Nevertheless, the GLFS-25 can identify individuals at high risk for falls in the coming year. Furthermore, the GLFS-25 score may predict the number of falls within a month, as shown in Table 1. This can enable preventive actions by families and interventions by public health experts. Despite these limitations, our findings provide important evidence of the relationship between GLFS-25 scores and falls.

## Conclusions

Our study showed that fallers had higher GLFS-25 scores, as well as higher body pain and anxiety scores, in comparison to non-fallers. Falls were linked to diagnoses of LS-1 or more, and a significant association existed between monthly fall frequency and GLFS-25 scores among residents aged ≥65 years. This emphasizes the importance of fall prevention in LS-1 individuals and suggests that the GLFS-25 total score may predict recurrent falls. Our study provides valuable evidence regarding the relationship between the GLFS-25 (total score and domain scores) and falls (both the history and the frequency of falls). However, the annual occurrence of falls was found to have no correlation with anything other than the GLFS-25 domain score related to physical pain and anxiety. Therefore, further investigations are needed.
